# Specific biological responses of the synovial membrane to carbon nanotubes

**DOI:** 10.1038/srep14314

**Published:** 2015-09-21

**Authors:** Hiroki Nomura, Seiji Takanashi, Manabu Tanaka, Hisao Haniu, Kaoru Aoki, Masanori Okamoto, Shinsuke Kobayashi, Takashi Takizawa, Yuki Usui, Ayumu Oishi, Hiroyuki Kato, Naoto Saito

**Affiliations:** 1 Department of Orthopaedic Surgery, Shinshu University School of Medicine, Asahi 3-1-1, Matsumoto 390-8621, Japan; 2 Institute for Biomedical Sciences, Interdisciplinary Cluster for Cutting Edge Research, Shinshu University, Asahi 3-1-1, Matsumoto 390-8621, Japan; 3 Aizawa Hospital Sports Medicine Center, Honjou 2-5-1, Matsumoto 390-8510, Japan

## Abstract

Biological evaluation of carbon nanotubes (CNTs) is typically performed in the lung or abdominal cavity; however, biological reactions to CNTs are predicted to be markedly different in other tissues. In applications of CNTs as reinforcement for artificial joints and drug delivery systems, including their use in bone regeneration, the intra-articular synovial membrane makes contact with the CNTs. Herein, we analyzed the reaction of the synovial membrane with multiwalled CNTs (MWCNTs). Injection of MWCNTs into rat knee joints revealed their dose-dependent incorporation into deep synovial membranes and the formation of granulation tissue, without long-term inflammation. MWCNTs were incorporated into human fibroblast-like synoviocytes (HFLSs), with less cytotoxicity than that observed in macrophages (RAW264 cells). Moreover, MWCNTs inhibited the release of cytokines and chemokines from HFLSs. The reaction of the synovial membrane with MWCNTs differed from that observed in other tissues; thus, detailed biological evaluation at each target site is necessary for clinical applications.

Biological reactions of carbon nanotubes (CNTs) have primarily been studied by subjecting test animals to inhalation of CNTs and then measuring their adhesion to the lung along with the resulting inflammatory reactions^1^. Abdominal cavity injection has also been performed to study the mechanism of mesothelioma occurrence because mesothelial tissue is common to both the lungs and abdominal cavity^2^. *In vitro* studies have mainly employed alveolar epithelial cells or macrophages, which are found in lung tissue^3^. However, there have been a growing number of studies on CNTs as biomaterials, highlighting the potential applications of these materials in drug delivery and imaging for cancer treatment, scaffolding for regenerative medicine, or combined with conventional implants as a reinforcement material^4^,^5^,^6^,^7^. The most important issue in such application of CNTs is the biological response of CNTs when embedded in tissue. When considering the specificity of CNT function in tissues or cells, biological reactions of CNTs have been shown to differ between embedded and inhaled CNTs. Moreover, it is necessary to evaluate the biological reactions of CNTs in various tissues, because their reactions are likely different^8^.

Studies of CNTs as a scaffold for bone tissue regeneration are very active^9^,^10^, and the combined use of CNTs with conventional materials, such as collagen, polymers, and hydroxyapatite, is expected. In the case of regeneration of intra-articular bone, CNT composites in the joint can cause intra-articular exposure of CNTs. Studies are underway in which CNTs are used as a reinforcement material for polyethylene in the sliding portion of artificial joints in order to improve wear resistance and to develop a long-lasting artificial joint^5^. When CNTs are used as a drug delivery system (DDS) for joint diseases, including rheumatoid arthritis and suppurative arthritis, CNT particles are administered directly into the joint^11^, and the intra-articular reaction of CNTs directly influences the efficacy of DDS. For the above reasons, the intra-articular reaction of CNTs should be further studied.

All joints are enclosed in an articular capsule, the inner surface of which is covered by the synovial membrane. Biological reactions, such as inflammation, mainly occur in the synovial membrane, as has been shown in studies of rheumatoid arthritis pathology and reactions to wear debris of artificial joints. Thus, the biological reaction of intra-articular CNTs must be evaluated, with focus primarily on the synovial membrane^12^,^13^.

Therefore, in this study, we investigated the biological reactions of MWCNTs in joints by evaluating the synovial tissue of animals and cultured synovial cells. To the best of our knowledge, this is the first study to describe the biological roles of MWCNTs in such a model; our data are expected to provide important insights into the applications of MWCNTs in the clinical setting.

## Results

### Setting the dosage of MWCNTs in the joint

MWCNTs with a mean diameter of 60 nm, length of 10 μm, and carbon purity of 99.5% or more were used in this experiment. For animal testing, 10-week-old male Wistar rats were used. MWCNTs were diffused in polysorbate 80, and the maximum feasible concentration was 2 mg/mL. Three concentrations of MWCNTs (0.02, 0.2, and 2 mg/mL) were prepared and injected (150 μL) into the rat knee. Thus, the weights of injected MWCNTs were 0.003, 0.03, and 0.3 mg, respectively. For comparison, a nanosized carbon material, Carbon Black (CB), which has a mean diameter of 47 nm, was used.

### Single intra-articular injection of MWCNTs

**Figure 1:**
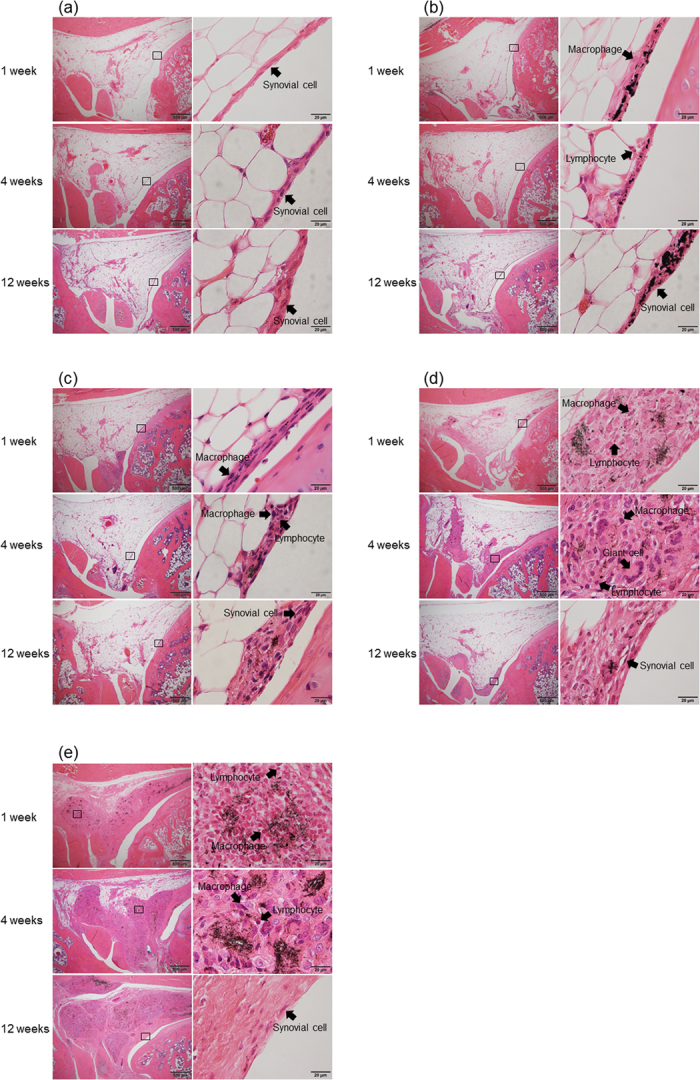
Analysis of the effects of a single intra-articular dose of MWCNTs using hematoxylin-eosin staining. (**a**) Histological images of the control group captured at 1, 4, and 12 weeks after physiological saline injection. Superficial tissue and deep adipose cells of synovial tissue were normal at all time points, and no inflammatory response was observed. (**b**) Histological images of 0.3 mg carbon black (CB) injection. After 1 week, CB invaded the synovial surface and was incorporated by macrophages, and a mild inflammatory response was observed primarily in the lymphocytes. CB was not observed in adipose tissue. After 4 weeks, the inflammatory response was improved, although CB was still incorporated in macrophages. At 12 weeks, the inflammatory response was resolved. (**c**) Histological images of 0.003 mg MWCNTs into the knee joint. After 1 week, MWCNTs invaded the synovial surface, which became mildly thickened. MWCNTs were incorporated into macrophages, and a mild inflammatory response was observed primarily in lymphocytes. After 4 weeks, the inflammatory response was alleviated, although MWCNTs were still incorporated in macrophages. At 12 weeks, the surface was covered with normal synovial tissue. (**d**) Histological images of 0.03 mg MWCNTs into the knee joint. After 1 week, MWCNTs had invaded the deep synovial tissue, and inflammatory cells (macrophages and lymphocytes) had replaced a portion of the adipose tissue. MWCNTs were aggregated and incorporated into macrophages. After 4 weeks, the inflammatory area was reduced, and a multinucleated foreign body giant cell made of fused macrophages was observed. After 12 weeks, the inflammatory response was resolved, and granulation tissue was formed in the normal synovial cells. (**e**) Histological images of 0.3 mg MWCNTs. MWCNTs invaded a larger area than that of the 0.03 mg group; however, no severe inflammatory response was observed. The inflammatory response was reduced after 4 weeks and resolved after 12 weeks. Granulation tissue was formed in a wider area than that in the 0.03 mg group; however, the synovial surface was covered with normal cells.

MWCNTs, CB, or saline (control) were injected into the front knee of rats as described in the Methods section. Rats were euthanized and, after observation of the whole synovial membrane, the synovial tissue (surface tissue and deep adipose tissue) around the patellar tendon attachment with the strongest tissue reaction was evaluated. In the control group, normal synovial tissue with a synovial surface was observed, and no inflammatory response was found (Fig. 1a).

One week after injection of CB, the synovial surface was invaded by CB and mildly thickened. CB was incorporated by macrophages, and a mild inflammatory response mainly involving the lymphocytes was observed; however, CB was not observed in deep adipose tissue. After 4 weeks, the inflammatory response was improved, the incorporation of CB to macrophages was maintained, and the thickened synovial membrane was unchanged. After 12 weeks, the inflammatory response had resolved (Fig. 1b).

The synovial surface was invaded by the MWCNTs and mildly thickened 1 week after administration of 0.003 mg of MWCNTs. MWCNTs were also incorporated by macrophages, and a mild inflammatory response (lymphocytes) was observed. Normal synovial fibroblasts were observed at the uppermost layer of the synovial surface. After 4 weeks, the inflammatory response was improved, and MWCNTs remained incorporated; after 12 weeks, normal synovial fibroblasts covered the uppermost layer (Fig. 1c).

One week after injection with 0.03 mg MWCNTs, the particles had invaded deep into the synovial tissue, and inflammatory cells had replaced part of the adipose tissue. No severe inflammatory responses were observed. MWCNTs were aggregated and incorporated by macrophages. After 4 weeks, the inflammatory area was gradually improved, and the surrounding area had become fibrotic. A multinucleated giant cell made of fused macrophages was observed. After 12 weeks, the inflammatory reaction was resolved, and granulation tissue had formed in the normal synovial cells (Fig. 1d).

One week after injection with 0.3 mg MWCNTs, invasion of a larger area than that of the 0.03 mg group was observed; however, invasion of leukocytes and severe inflammatory responses were not observed. After 4 and 12 weeks, the inflammatory response improved with time, and granulation tissue was formed over a wide area; however, the synovial surface was covered with normal cells (Fig. 1e).

### Comparison of single- and multiple-dosing regimens for MWCNTs

Multiple intra-articular administrations are likely to be performed when MWCNTs are used in DDS, and gradual intra-articular release of MWCNTs caused by wear is expected when used in artificial joints. Therefore, it is necessary to evaluate the differences in synovial tissue responses using single- or multiple-dosing regimens for MWCNTs. To evaluate the differences, 0.003 mg of MWCNTs was administered either through a single dose or through three doses (0.001 mg each) with a 1-week interval, and the synovial response was evaluated 1 and 4 weeks after administration of the final dose. As a control, physiological saline was also administered over a 1-week interval.

**Figure 2:**
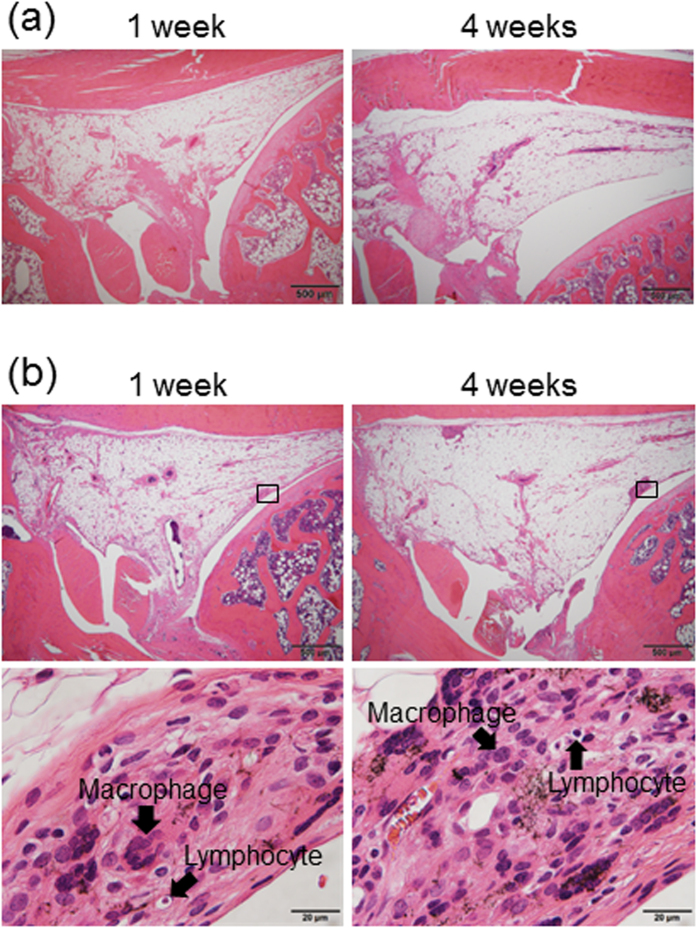
Evaluation of equivalency between multiple and single administrations of MWCNTs by hematoxylin-eosin stain. (**a**) Synovial histological images captured 1 and 4 weeks after administration of 150 μL physiological saline to rat knee joints three times with a 1-week interval (control group). Normal synovial tissue, similar to that in the single-dose group, was observed. (**b**) Synovial histological images captured 1 and 4 weeks after administration of 0.003 mg MWCNTs (0.001-mg doses three times with a 1-week interval) to the rat knee joint. After 1 week, MWCNTs had invaded the synovial surface and were incorporated by macrophages. A mild inflammatory response, which was similar to that at 1 week after a single 0.003 mg MWCNT dose, was observed. One week after the third administration of MWCNTs, the synovial surface was mildly thickened, similar to that observed after the single dose. The inflammatory response was resolved after 4 weeks, and results were similar to those after a single administration.

The control group showed normal synovial tissue, similar to that of the single administration group, after 1 and 4 weeks of the final dose (Figs 1a and 2a).

One week after the final dose in the group with multiple administrations of MWCNTs, the synovial surface was invaded by MWCNTs and mildly thickened, with a mild inflammatory response and MWCNTs incorporated in macrophages. The histological image captured was almost identical to that after the single dose. Four weeks after the three administrations of MWCNTs, the inflammatory response was milder than that after 1 week, the synovial surface was covered with normal synovial cells, and the histological image captured was similar to that after the single administration (Figs 1c and 2b). Based on these results, we concluded that the effects of multiple intra-articular administrations of MWCNTs on synovial tissue were similar to those of a single administration.

### Incorporation of MWCNTs and CB into cells

**Figure 3:**
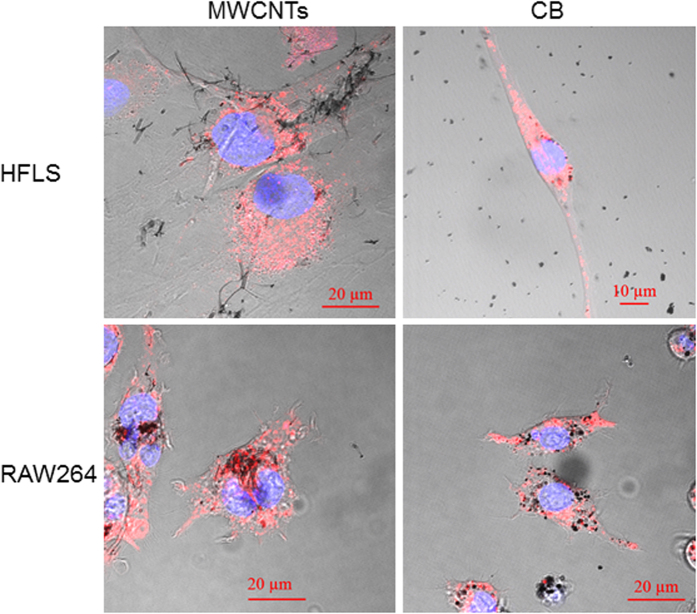
Incorporation of MWCNTs into cells. Human fibroblast-like synoviocytes (HFLSs) and RAW264 cells were cultured and exposed to either 10 μg/mL MWCNTs or CB for 24 h. Both HFLSs and RAW264 cells exhibited incorporation of MWCNTs and CB, with RAW264 cells showing increased incorporation per cell. Both MWCNTs and CB were observed in lysosomes of HFLSs and RAW264 cells. Blue: nucleus, Red: lysosome.

Human fibroblast-like synoviocytes (HFLSs) were used as synovial cells. As a comparison, RAW264 cells, a mouse macrophage generally used in CNT cell studies, were used. These cells were exposed to 10 μg/mL of MWCNTs or CB for 24 h, and staining solutions (for lysosome and cell nucleus) were added to observe cell morphology as well as incorporation and localization of MWCNTs and CB within the cells. Cells were cultured and treated as described in the Methods section. Incorporation of both MWCNTs and CB into the HFLSs and RAW264 cells was observed; however, there was no change in cell morphology. HFLSs showed less incorporation per cell compared with RAW264 cells for both MWCNTs and CB. Both MWCNTs and CB existed in lysosomes (stained red) in HFLSs and RAW264 cells (Fig. 3).

### Influence of MWCNTs and CB on cell proliferation

In cell proliferation tests, we analyzed the effects of MWCNTs and CB at concentrations of 0.1, 1, 10, and 100 μg/mL, in reference to previous CNT cell proliferation tests^14^, and Alamar Blue was used for staining. Cells were cultured and treated as described in the Methods section.

**Figure 4:**
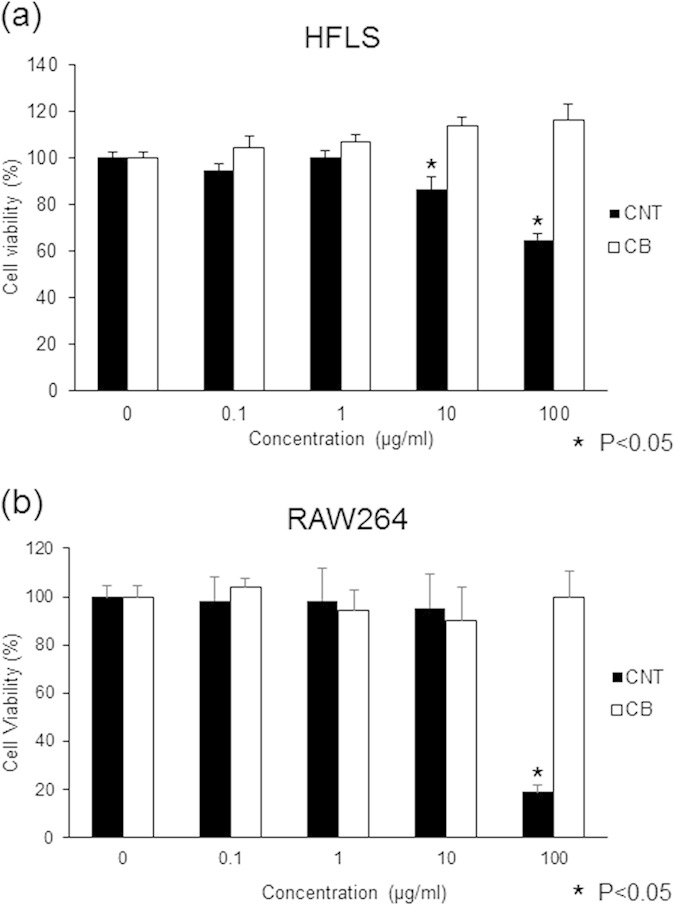
Proliferation of HFLSs and RAW264 cells exposed to MWCNTs. Cultured HFLSs and RAW264 cells were exposed to 0, 0.1, 1, 10, or 100 μg/mL of MWCNTs or CB. After 24 h of exposure, Alamar Blue staining was performed, and cell numbers were counted 4 h later. (**a**) In HFLS cells, CB did not result in inhibition of proliferation at any concentration. MWCNTs resulted in a dose-dependent decrease in cell proliferation at 10 and 100 μg/mL at rates of 86% and 64% that of the control, respectively. The difference was statistically significant. (**b**) For RAW264 cells, CB did not result in inhibition of proliferation at any concentration. When treated with 100 μg/mL MWCNTs, cell proliferation was inhibited at a rate of 18% that of the control. The difference was statistically significant.

In HFLSs, cell proliferation was not inhibited at all CB concentrations. In contrast, treatment with MWCNTs at 10 or 100 μg/mL led to a significant, dose-dependent decrease in cell proliferation (86% or 64%, respectively, relative to the control group; Fig. 4a).

In RAW264 cells, proliferation was also not inhibited in all CB groups. However, the proliferation of cells treated with the highest MWCNT concentration (100 μg/mL) was decreased significantly, reaching 18% that of the control (Fig. 4b).

### Release of cytokines and chemokines

Cell proliferation tests showed that cell proliferation was strongly inhibited after treatment with 100 μg/mL MWCNTs, which would prevent the measurement of cytokine and chemokine secretion; thus, evaluation was carried out at concentrations of 0, 0.1, 1, and 10 μg/mL MWCNTs or CB for 24 h. Cells were then pelleted by centrifugation, and the supernatants were collected. The cytokines and chemokines measured in this study have been reported to play a role in wear debris-related osteolysis^15^. HFLSs secreted interleukin (IL)-6, IL-8, and monocyte chemotactic protein (MCP)-1, while RAW264 cells secreted tumor necrosis factor (TNF)-α, MCP-1, macrophage inflammatory protein (MIP)-1α, and regulated on activation, normal T cell expressed and secreted (RANTES).

**Figure 5:**
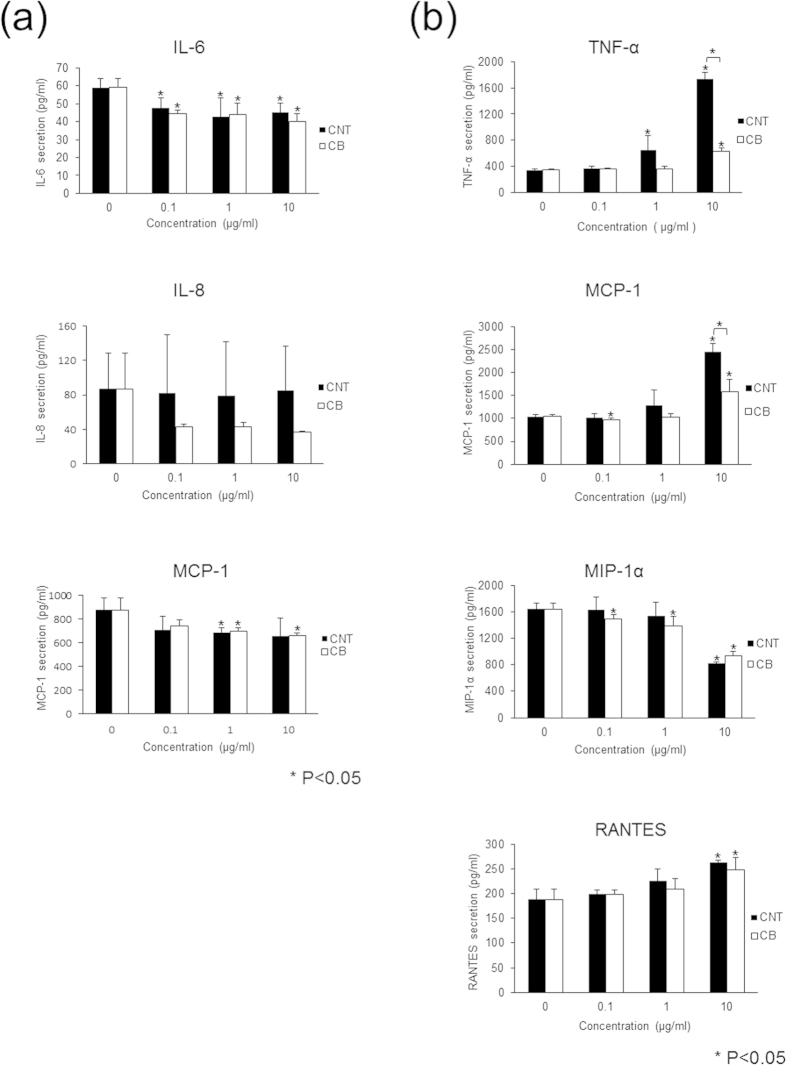
Cytokine and chemokine secretion from HFLSs and RAW264 cells exposed to MWCNTs. Cultured HFLSs and RAW264 cells were exposed to 0, 0.1, 1, or 10 μg/mL MWCNTs or CB. After 24 h of exposure, detection and measurement of cytokines and chemokines were performed. (**a**) HFLSs secreted IL-6, IL-8, and MCP-1. IL-6 secretion was reduced by both MWCNTs and CB exposure, but the dose-dependent effects were not clear. IL-8 was unchanged by MWCNTs and showed a decreasing tendency with CB, without dose dependency, and the difference was not significant. MCP-1 secretion was significantly reduced by 1 μg/mL MWCNTs and 1 or 10 μg/mL CB, but the effects were not dose dependent. (**b**) RAW264 cells secreted TNF-α, MCP-1, MIP-1α, and RANTES. TNF-α secretion was significantly increased by 1 or 10 μg/mL MWCNTs and 10 μg/mL CB, and MWCNTs resulted in a larger increase than did CB. MCP-1 secretion was increased by 10 μg/mL MWCNTs and CB, and MWCNTs resulted in a larger increase than did CB. MIP-1α secretion was significantly decreased by 10 μg/mL MWCNTs and was significantly decreased in response to CB in a dose-dependent manner. RANTES secretion was significantly increased by both 10 μg/mL MWCNTs and CB.

In HFLSs, secretion of IL-6 was significantly decreased with exposure to MWCNTs and CB, but the dose dependency of this effect was unclear. IL-8 secretion was unchanged by MWCNT exposure and decreased with CB exposure, regardless of the concentration; however, the decrease was not significant. MCP-1 was significantly decreased following exposure to 1 μg/mL MWCNTs and 1 or 10 μg/mL CB, but this effect was not dose dependent (Fig. 5a). Therefore, exposure to MWCNTs tended to decrease cytokine and chemokine secretion in HFLSs, but without a clear dose-dependent effect.

In RAW264 cells, TNF-α secretion was significantly increased by 1 or 10 μg/mL MWCNTs and 10 μg/mL CB exposure. The increase in TNF-α secretion by MWCNTs was larger than that by CB. MCP-1 secretion was significantly increased by 10 μg/mL MWCNTs and CB, and the increase induced by MWCNTs was larger than that induced by CB. MIP-1α secretion was significantly decreased by exposure to 10 μg/mL MWCNTs, and CB exposure caused a significant dose-dependent decrease in MIP-1α secretion. RANTES secretion was significantly increased with exposure to either 10 μg/mL MWCNTs or CB (Fig. 5b). Overall, MWCNTs increased the secretion of many cytokines and chemokines in RAW264 cells in a dose-dependent manner, and only MIP-1α exhibited decreased secretion.

## Discussion and Conclusions

In this study, we examined the reaction of the synovial membrane MWCNTs. Injection of MWCNTs into rat knee joints revealed dose-dependent incorporation into deep synovial membranes and formation of granulation tissue, without long-term inflammation. MWCNTs were incorporated into HFLSs and RAW264 cells and inhibited the release of cytokines and chemokines from HFLSs. Thus, our data demonstrated that detailed biological evaluation of the reactions of MWCNTs at each target site is necessary for clinical applications of these biomaterials.

Animal tests in this study showed a highly specific reaction to MWCNTs in the synovial membrane. MWCNTs invaded deep tissue from the synovial surface and were then incorporated by macrophages to form granulation tissue, which replaced the original adipose tissue and was stable over time. The amount of granulation tissue within the adipose tissue was dependent on the amount of MWCNTs. At the smallest amount of MWCNTs used in this study, granulation tissue was limited to the synovial surface; when the amount of MWCNTs was increased by 100-fold, a wide area of adipose tissue was replaced by granulation tissue. In contrast, when CB was injected into the joint at the same dose at which MWCNTs formed a large amount of granulation tissue, only a small amount of CB was incorporated into the synovial surface, and CB did not invade into the deep adipose tissue. MWCNTs and CB are both nanosized carbon particles, but the invasiveness of these two types of particles was highly different, presumably because of the different morphologies of these particles. MWCNTs, which have a fibrous shape, may be entangled with the pleated synovial membrane and incorporated to a greater extent than CB, which has a spherical shape. In addition, sphere-shaped CB may stay at the synovial surface, while the fibrous MWCNTs pass through the synovial surface and reach the deep adipose tissue to form granulation tissue. Frequent and active movement of the knee joint may facilitate this transportation. The loose connection of the synovial surface and the absence of desmosome formation may strongly support the above hypothesis^16^. The large difference in reaction to the synovial membrane between these nanosized carbon particles is very interesting. Because only a few nanosized biological materials have been described to date, the responses of various tissues to these particles have not yet been defined. An even wider variety of tissue responses to nanosized biological materials will likely be identified with the development of more materials in the future.

The morphology of the synovial surface tissue remained normal after a large amount of MWCNTs had incorporated into deep tissue. Granulation tissue in deep tissue was formed from both macrophages incorporating MWCNTs and fibrous tissue, and the inflammatory response had resolved in 4 weeks. Interestingly, in this study, we observed a milder inflammatory response than what is commonly observed. The inflammatory response induced by CB in the synovial surface was also milder than what is typically observed. From these results, we concluded that nanosized carbon particles did not cause a strong inflammatory response in the synovial surface and deep tissue.

From the equivalency test of multiple and single doses of MWCNTs, the synovial histological image captured after three divided doses to the knee joint was similar to that taken after a single dose. This result suggested that single-dose animal testing can be employed to evaluate the synovial tissue response to MWCNTs intermittently released in clinical settings. This finding is valuable because safety evaluations of MWCNT implants, which release MWCNTs gradually, or MWCNT DDSs requiring multiple injections to the knee can be performed using a single-dose test.

Aggregation of nanoparticles in the dilution can affect biological reactions in animal tests. Although MWCNTs and CB are both carbon nanoparticles having similar diameters, the aggregated secondary particles injected into the joints have different diameters because of the differences in morphology (see Supplementary Information). In addition, the diameter of the secondary particles is likely to change over time, and it is difficult to judge the influence of differences in diameter on the synovial inflammatory reactions. Further studies are required to determine the relationship between the secondary diameter of nanoparticles and biological reactions.

RAW264 cells have been reported to incorporate MWCNTs and CB^17^,^18^; however, HFLSs have also been shown to incorporate these materials, albeit at lower concentrations. Synovial cells have been classified as A-type cells, similar to phagocytes, e.g., macrophages and fibroblast-like B-type cells; however, recent studies have also described C-type cells, which exhibit an intermediate phenotype between A- and B-type cells. The function and morphology of the synovial membrane can be altered by various stimuli and environments^16^,^19^,^20^,^21^. The HFLSs used in this study have phagocytic function; thus, these cells are not expected to resemble pure fibroblast-like B-type cells.

Cell proliferation tests showed that a high concentration of MWCNTs decreased the proliferation of both HFLSs and RAW264 cells, while the same concentration of CB did not. Various studies have examined the inhibition of RAW264 cell proliferation by MWCNTs, including the influence of phagocytosis^22^,^23^. In this study, we showed that HFLS proliferation was inhibited slightly by 10 μg/mL MWCNTs and to 64% at 100 μg/mL MWCNTs, which was lower than that observed in RAW264 cells (18% inhibition at 100 μg/mL).

In the measurement of cytokines and chemokines in RAW264 cells, MWCNTs increased the TNF-α-, MCP-1-, and RANTES-inducing inflammatory responses in a dose-dependent manner. In contrast, the secretion of MIP-1α was decreased in a dose-dependent manner. However, the mechanism mediating this effect is unclear. In HFLSs, MWCNTs significantly decreased the secretion of IL-6 and MCP-1. If the mild inflammation that was quickly resolved in the synovial membrane after injection of large amounts of MWCNTs could be attributed to the inhibitory effects of MWCNTs on cytokine and chemokine release from the synovial membrane, these findings would be very important and provide novel insights into the mechanisms of MWCNT function. The absence of a dose-dependent effect suggested the possibility that the inhibition occurred at a lower concentration than tested or that the inhibition could be attributed to a different factor; thus, further studies of the mechanism are warranted.

The amount of MWCNTs injected in this study was based on the maximum possible dose in order to evaluate the risk of injecting MWCNTs into the joint. The results provide indications for the clinical use of MWCNTs in the joint as a biomaterial. If human and rat weights are assumed to be 60 and 0.250 kg, respectively, the weight of MWCNTs injected in rats (0.003, 0.03, and 0.3 mg) is equivalent to injection of 0.72, 7.2, and 72 mg in humans. It is difficult to determine the clear safety threshold of MWCNTs in human intra-articular synovial tissue; however, it is necessary to establish a standard dose for intra-articular use of MWCNTs. Our study suggested that administration of 0.003 mg MWCNTs in rats, which is equivalent to 0.72 mg in humans, is a safe dose because MWCNTs stay on the synovial surface. After administration of 0.03 mg MWCNTs in rats, equivalent to 7.2 mg in humans, only a small amount of MWCNTs invaded into the deep tissue, and the inflammatory reaction was ameliorated within a short time; thus, safety could be expected. The formation of granulation tissue within the deep tissue following administration of the equivalent of 72 mg MWCNTs or more in humans suggested that this the amount was too high. However, the maximum dose used in this study is unlikely to be used clinically.

The influence of MWCNTs on articular cartilage was not evaluated in this study, because it is not relevant in artificial joints, in which the cartridge is resected. However, sufficient evaluation is necessary for application of DDSs in rheumatoid arthritis. Importantly, MWCNTs have been shown to have high biocompatibility in bone tissues^9^,^24^,^25^.

In summary, this study could serve as a model for evaluating the biological response to CNTs in specific physiological systems. The response of the synovial membrane in the joint to MSCNTs was found to be different from that in the lung or abdominal cavity. Currently, many research groups are studying the biological application of CNTs in cancer treatment and regenerative medicine^26^,^27^,^28^, and additional evaluation of safety is critical for the clinical application of CNTs based on these studies^8^. The response of the lung or abdominal cavity to CNTs is generally thought to be representative of the biological reaction; however, these findings are only applicable to inhalation because the response to implantation of CNT-combined biomaterials is completely different. In this study, we demonstrated the importance of such a difference and revealed the necessity for strict evaluation of biological responses within specific sites of application. We believe the findings of this study provide important insights into the biological evaluation of CNTs for combined application of CNTs with biomaterials.

## Methods

### MWCNTs

MWNT7 (Hodogaya Chemical Co., Ltd., Tokyo, Japan) with a mean diameter of 60 nm, length of 10 μm, and carbon purity of 99.5% or more was used^29^. The dispersing agent was polysorbate 80 (NOF Corporation, Tokyo, Japan) diluted to 0.1% in phosphate-buffered saline (PBS; Gibco, NY, USA).

### CB

The nanosized carbon material CB (Mitsubishi Chemical Corporation, Tokyo, Japan), with a mean diameter of 47 nm, was used for comparison after dispersal in 0.1% in polysorbate 80 (2 mg/mL dispersion).

### Selection of MWCNT dose for injection

Ten-week old male Wistar rats (Japan SLC, Shizuoka, Japan) were used for animal testing. The maximum volume of liquid able to be injected into the unilateral knee joint without leakage was determined to be 150 μL. The maximum concentration of MWCNTs that could be injected by a 25-gauge needle was 2 mg/mL, and the concentrations of MWCNTs injected were 0.02, 0.2, and 2 mg/mL, equal to MWCNT weights of 0.003, 0.03, and 0.3 mg, respectively. All animal experiments complied with the guidelines of the Institutional Animal Care Committee of Shinshu University.

### Rat single intra-articular administration test

Rats were sedated by inhalation of isoflurane (3.0%, 2.0 mL/min; Abbott Japan Co., Ltd., Tokyo, Japan). An 8-mm median incision was made to cut the synovial capsule, and the tibial tuberosity and patellar tendon were exposed. Injections (150 μL each) to the joint were made with a 1-mL syringe and 25-gauge needle. Pressure was then applied to the site with gauze to prevent outflow from the joint, and the incision site was sutured with 5–0 nylon thread. Rats were euthanized 1, 4, or 12 weeks after injection using a high concentration of isoflurane, and the knee joint was resected. The surrounding soft tissue was removed, and tissues were fixed in formalin (Wako Pure Chemical Industries, Ltd., Osaka, Japan) for 2 weeks. Decalcification was then performed for 48 h using K-CX (Falma, Tokyo, Japan), thin sections were prepared, and hematoxylin and eosin staining was performed. Tissues were evaluated with an optical microscope.

### Multiple administrations of MWCNTs in rats

MWCNTs (0.003 mg) were administered via three doses (0.001 mg per dose, 1-week interval) to the rat knee joint. As a control, physiological saline (150 μL) was also injected with a 1-week interval. Rats were euthanized 1 or 4 weeks after the final dose, and the knee joint was resected. Preparation and evaluation of tissue sections were performed as described for the single-dose experiment.

### Cell culture

HFLS cells (Lot No. 2924; Cell Application, CA, USA) were cultivated using a synoviocyte growth medium kit (Cell Application) in 10-cm petri dishes. Culture medium was replaced daily until cells were about 60% confluent. Macrophages (Lot No. 36; RAW264 cells; Riken, Saitama, Japan) were used for comparison. Basic culture, including MEM medium (Wako Pure Chemical Industries, Ltd., Osaka Japan), 1% glutamine acid (Sigma-Aldrich, MO, USA), and 10% fetal bovine serum (FBS; Gibco), was used for continuous culture. Both HFLSs and macrophages were continuously cultured to keep the cell number at approximately 4.0 × 10^5^ cells. Cells before the sixth passage were used for further analysis.

### Incorporation of MWCNTs into cells

For morphological observation, HFLSs and RAW264 cells were cultured in 8-well chamber slides at 4.0 × 10^4^ cells/well. The culture medium was removed after 24 h, and cells were exposed to 10 μg/mL MWCNTs or CB. After 24 h, cells were rinsed twice with 200 μL prewarmed (37 °C) PBS, and 100 μL of culture medium was then added. Staining solution, containing a mixture of lysosome stain (Cyto Painter Lysosomal Staining ab 138895; Abcam, Cambridge, UK) and nuclear stain (Bisbenzimide H33342 Fluorochrome Trihydrochloride DMSO Solution; Nacalai Tesque, Inc., Kyoto, Japan), was added, and observed after 3 h using a microinjection apparatus (Carl Zeiss, Oberkochen, Germany).

### Effect of MWCNTs and CB on cell proliferation

Cells were cultured at 1.0 × 10^4^ cells/well in 96-well plates. After 24 h, the culture medium was removed, and cells were exposed to MWCNTs or CB at concentrations of 0.1, 1, 10, or 100 μg/mL (six samples per concentration). After 24 h of exposure, cells were stained with Alamar Blue (Invitrogen Life Technologies, Tokyo, Japan) for 4 h, and cell numbers were determined using a microplate reader (Biotech Japan Corporation, Tochigi, Japan). The results were analyzed to determine significant differences as described below.

### Detection and measurement of cytokines and chemokines

HFLSs were cultured at a density of 1.25 × 10^4^ cells/well, and RAW264 cells were cultured at a density of 2.5 × 10^4^ cells/well. After 24 h, the culture medium was removed, and cells were exposed to 0.1, 1, or 10 μg/mL MWCNTs or CB. After 24 h, samples were centrifuged to separate sediment, and the supernatant was collected carefully so as not to absorb MWCNTs or CB. Detection and measurement of cytokines and chemokines were performed using a Cytometric Bead Array System (Becton Dickinson, NJ, USA) with inflammation and chemokine kits.

### Statistics

Student’s *t*-tests were used for statistical analysis, and differences with *p* values of 0.05 or less were considered statistically significant.

### Ethics

All experimental protocols were approved by the Institutional Animal Care Committee of Shinshu University. The methods were carried out in accordance with approved guidelines.

## Additional Information

**How to cite this article**: Nomura, H. *et al.* Specific biological responses of the synovial membrane to carbon nanotubes. *Sci. Rep.* **5**, 14314; doi: 10.1038/srep14314 (2015).

## Supplementary Material

Supplementary Information
