# Supplementary material for: Specific biological responses of the synovial membrane to carbon nanotubes
Source: Sci Rep. 2015 Sep 21;5:14314. doi: 10.1038/srep14314 (PMC4585697; doi:10.1038/srep14314)
Supplement: Supplementary Information [file srep14314-s1.pdf]

Supplementary information

Specific biological responses of the synovial membrane to carbon nanotubes

Hiroki Nomura[1], Seiji Takanashi[1], Manabu Tanaka[1], Hisao Haniu[2], Kaoru Aoki[1], Masanori Okamoto[1], Shinsuke Kobayashi[1], Takashi Takizawa[1], Yuki Usui[3], Ayumu Oishi[2], Hiroyuki Kato[1] and, Naoto Saito[2]*

[1]Department of Orthopaedic Surgery, Shinshu University School of Medicine, Asahi 3-1-1, Matsumoto 390-8621, Japan

[2]Institute for Biomedical Sciences, Interdisciplinary Cluster for Cutting Edge Research, Shinshu University, Asahi 3-1-1, Matsumoto 390-8621, Japan

[3]Aizawa Hospital Sports Medicine Center, Honjou 2-5-1, Matsumoto 390-8510, Japan

*Corresponding author:

Naoto Saito, Professor & Director, Institute for Biomedical Sciences, Interdisciplinary Cluster for Cutting Edge Research, Shinshu University, Asahi 3-1-1, Matsumoto, 390-8621 Japan; TEL, FAX: +81 263 37 2409, e-mail: saitoko@shinshu-u.ac.jp

Supplementary Materials and Methods

Average diameters of aggregated secondary particles of MWCNT7 (Hodogaya Chemical Co., Ltd., Tokyo, Japan) and Carbon Black (CB; Mitsubishi Chemical Corporation, Tokyo, Japan) were measured. Dispersion liquid was prepared by diluting polysorbate 80 (NOF Corporation, Tokyo, Japan) in phosphate-buffered saline (PBS; Gibco, NY, USA) to 0.1%. MWCNTs and CB were diluted to 0.02 mg/mL, and the diameters were measured three times each using a Zetasizer Nano Range instrument (Malvern Instruments, Malvern, UK).

Supplementary Results

The average diameters of secondary particles for MWCNTs and CB were 1756 and 285 nm (Supplementary Figure).

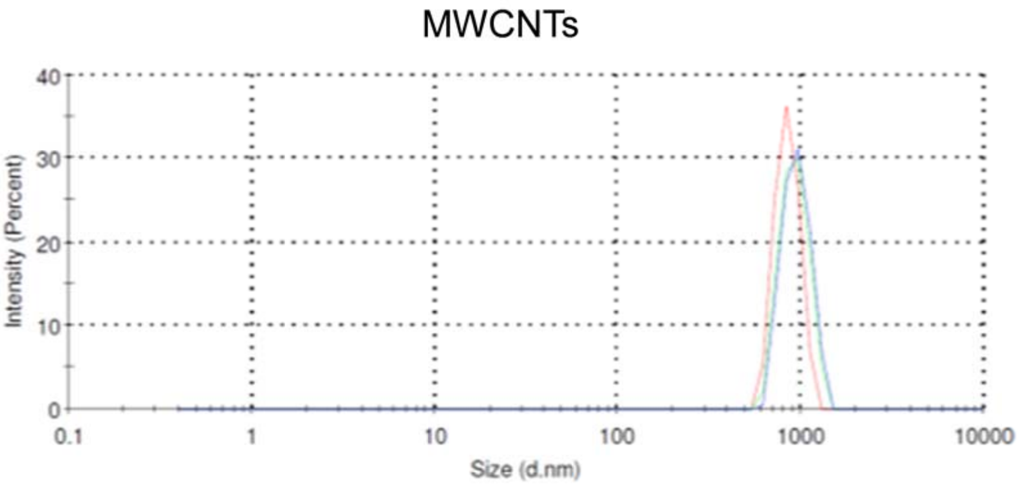

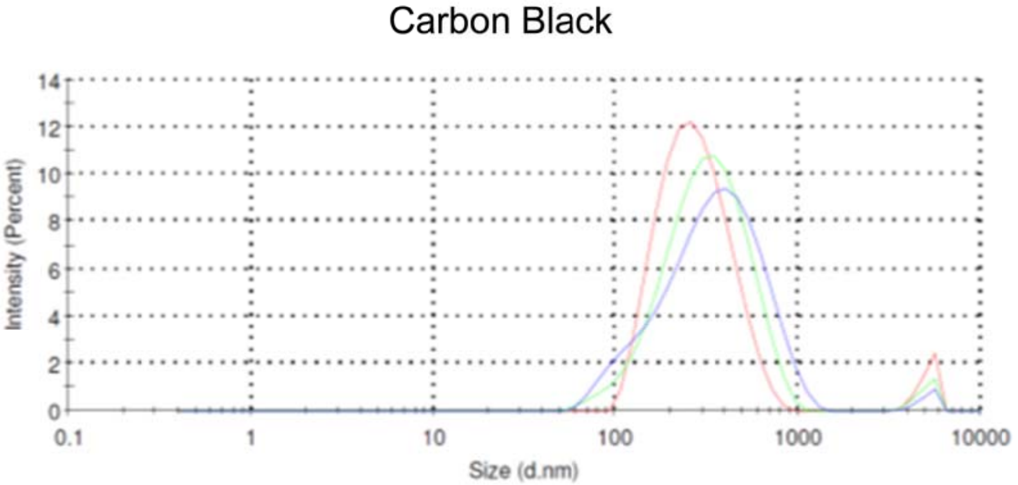

Supplementary Figure. Average diameter of secondary particles for MWCNTs and Carbon Black (0.02 mg/mL).
